# Anti-N-methyl-D-aspartate receptor encephalitis concomitant with multifocal subcortical white matter lesions on magnetic resonance imaging: a case report and review of the literature

**DOI:** 10.1186/s12883-015-0366-5

**Published:** 2015-07-08

**Authors:** Rui-Jin Wang, Bu-Dong Chen, Dong Qi

**Affiliations:** Department of Neurology, Beijing Friendship Hospital, Capital Medical University, No. 95 Yong-An Road, Xicheng District, Beijing, 100050 People’s Republic of China; Department of Radiology, Beijing Friendship Hospital, Capital Medical University, No. 95 Yong-An Road, Xicheng District, Beijing, 100050 People’s Republic of China

**Keywords:** Anti-N-methyl-D-aspartate receptor encephalitis, Subcortical white matter lesions, Plasma exchange, Magnetic resonance imaging

## Abstract

**Background:**

Anti-N-methyl-D-aspartate receptor encephalitis is a severe autoimmune disorder characterized by severe psychiatric symptoms, seizures, decreased consciousness, autonomic dysregulation, and dyskinesias. Multifocal subcortical white matter lesions on fluid-attenuated inversion recovery and diffuse weighted images have rarely been reported in previous literature, and serial magnetic resonance imaging changes after plasma exchange have not been presented before.

**Case presentation:**

A previously healthy 24-year-old Chinese woman presented with acute psychiatric symptoms characterized by fear and agitation followed by decreased consciousness, dyskinesias, and seizures. Magnetic resonance imaging revealed hyperintense lesions on fluid-attenuated inversion recovery and diffuse weighted images in bilateral subcortical white matter. Cerebrospinal fluid analysis revealed a mild pleocytosis with lymphocytic predominance. Protein and glucose levels were normal. Aquaporin-4 antibodies in serum and cerebrospinal fluid were negative. Identification of anti-N-methyl-D-aspartate receptor antibodies in serum and cerebrospinal fluid confirmed the diagnosis of anti-N-methyl-D-aspartate receptor encephalitis. She was initially treated with combined intravenous immunoglobulin and methylprednisolone without improvement. Plasma exchange was then initiated with good response; the patient made a full recovery after several cycles of plasma exchange. Repeat magnetic resonance imaging performed 1 month after plasma exchange showed partial resolution of the hyperintense lesions in bilateral subcortical white matter, and follow-up magnetic resonance imaging 2 months after plasma exchange showed complete resolution.

**Conclusion:**

Anti-N-methyl-D-aspartate receptor encephalitis may be concomitant with multifocal subcortical white matter lesions. Such lesions may resolve after appropriate immunotherapy.

## Background

Anti-N-methyl-D-aspartate (NMDA) receptor encephalitis is a severe autoimmune disorder first described by Dalmau and his colleagues in 2007 [1]. Patients with anti-NMDA receptor encephalitis usually have an acute onset. The typical clinical features include severe psychiatric symptoms, memory loss, seizures, decreased consciousness, autonomic dysregulation, and dyskinesias [2].

Here, we describe a case of anti-NMDA receptor encephalitis with multifocal subcortical white matter lesions on fluid-attenuated inversion recovery (FLAIR) and diffuse weighted images (DWI). This patient responded well to plasma exchange treatment. Serial magnetic resonance imaging (MRI) changes after this treatment are presented. To our knowledge, multifocal subcortical white matter lesions on FLAIR and DWI have rarely been described in previous literature, and serial MRI changes after plasma exchange have not been presented before.

## Case presentation

A 24-year-old Chinese woman presented with acute psychiatric symptoms characterized by fear and agitation. Two days after symptom onset, she was admitted to a psychiatric hospital, and her symptoms were temporarily controlled. Seven days after symptom onset, she developed a low-grade fever and decreased consciousness, at which point she was transferred to our hospital. Her past medical history was unremarkable. On examination, she was non-responsive to verbal or painful stimulation. Her vital signs indicated slightly elevated heart rate and body temperature and normal blood pressure and respiratory rate. The pupils were normal in size and reactive to light. Spontaneous eye movements were absent. The oculocephalic responses were present. Babinski’s sign was negative bilaterally, and both Kernig’s and Brudzinski’s signs were negative.

**Fig. 1:**
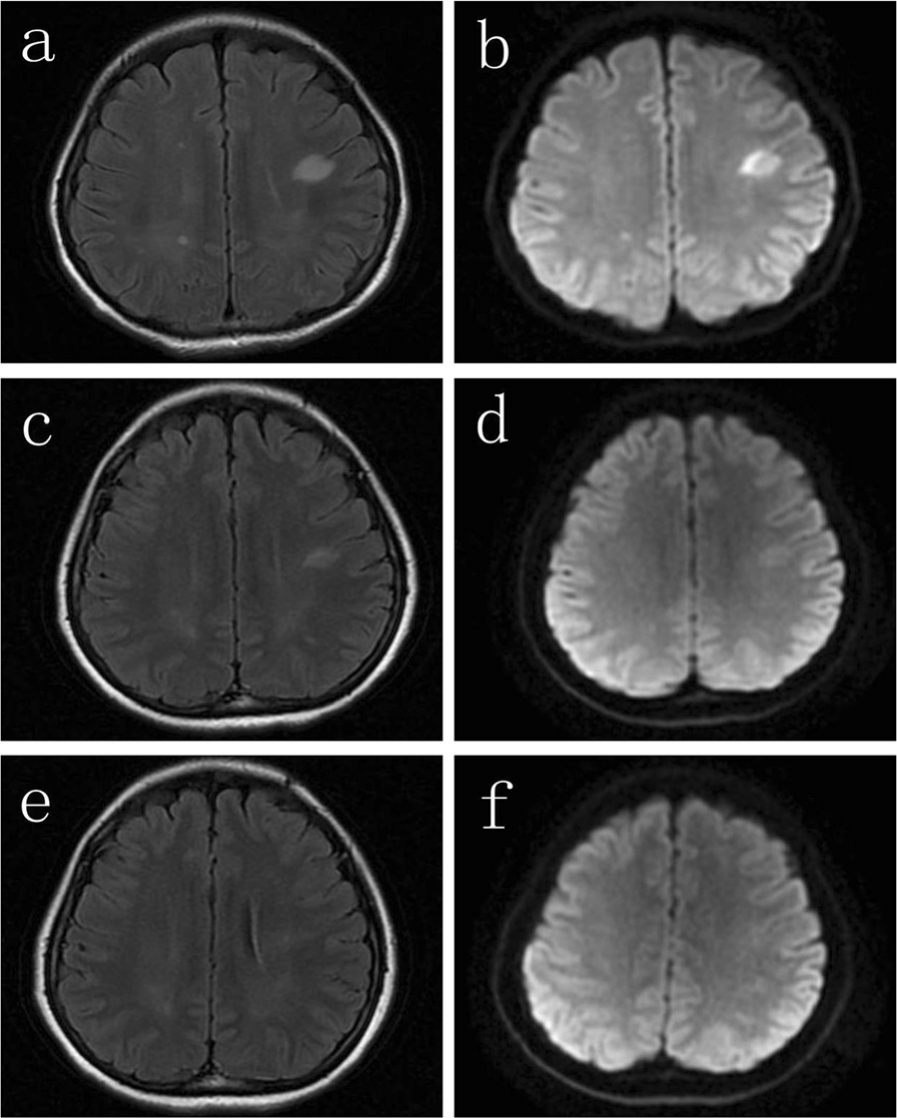
Serial MRI findings: hyperintense lesions on FLAIR and DWI in bilateral subcortical white matter before plasma exchange (**a**, **b**); partial resolution of the lesions on FLAIR and DWI 1 month after plasma exchange (**c**, **d**); full resolution of the lesions on FLAIR and DWI 2 months after plasma exchange (**e**, **f**)

Routine blood test and chemistry analysis including liver, renal, and thyroid function were unremarkable. The erythrocyte sedimentation rate was normal. Autoimmune markers including antinuclear antibodies, anti-double-stranded DNA (anti-dsDNA) antibodies, rheumatoid factor, and antineutrophilcytoplasmic antibodies (ANCA) were negative. Serum anti-thyroglobulin (anti-TG) antibodies (38.4U/ml, normal range 0–60 U/ml, radioimmunoassay) and anti-thyroid peroxidase (anti-TPO) antibodies (28 U/ml, normal range 0–60 U/ml, radioimmunoassay) were unremarkable. Brain computed tomography (CT) was normal. MRI revealed FLAIR and DWI hyperintense lesions in bilateral subcortical white matter (Fig. 1a,b). Electroencephalography (EEG) showed diffuse slow-waves. Cerebrospinal fluid (CSF) analysis revealed a mild pleocytosis (24 × 10^6^/L) with lymphocytic predominance. Protein and glucose levels were normal. Aquaporin-4 antibodies in serum and CSF were negative. Serological and polymerase chain reaction (PCR) investigation in serum and CSF were negative for infectious etiologies including varicella zoster virus, enterovirus, herpes simplex virus, cytomegalovirus, and Epstein-Barr virus. Bacterial cultures of the CSF were negative. Anti-NMDA receptor antibodies were identified both in serum (titers 1:100) and CSF (titers 1:16).

The diagnosis of anti-NMDA receptor encephalitis was established. Thorough evaluation, including ovarian ultrasound and high resolution computed tomography of the chest, abdomen, and pelvis, did not reveal an ovarian teratoma or other tumors. Intravenous immunoglobulin (IVIg, 0.4 g/kg/day for 5 days) combined with high dose methylprednisolone (1g/day for 5 days) was initiated on day 18 after symptom onset. Despite IVIg and steroid therapy, the patient’s level of consciousness did not improve. She developed dyskinesias with mouth chewing, tongue thrusting, and twisting of the upper limbs. Episodes of generalized tonic-clonic convulsions were also seen. Repeat EEG again revealed diffuse slow-waves. At this point, the patient was intubated and mechanically ventilated. Valproic acid and intermittent intravenous diazepam was administered to control the seizures. On day 35, plasma exchange therapy was initiated; the patient regained consciousness after two cycles of plasma exchange, and seizures and dyskinesias were controlled after three cycles of this treatment. An additional four cycles of plasma exchange were given to reinforce the efficacy. Repeat MRI performed 1 month after plasma exchange showed partial resolution of the hyperintense lesions in bilateral subcortical white matter on FLAIR and DWI (Fig. 1c,d). MRI performed 2 months after plasma exchange showed complete resolution (Fig. 1e,f). Repeat CSF analysis revealed normal leucocytes (2 × 10^6^/L), as well as normal protein and glucose levels. Anti-NMDA receptor antibodies were undetectable in serum and CSF. At 12-months follow-up, the patient remained well and has had no relapse of seizures and dyskinesias.

## Discussion

Our patient presented with acute psychiatric symptoms, followed by low-grade fever, decreased consciousness, dyskinesias, and seizures. This case demonstrates the classic clinical presentation of anti-NMDA receptor encephalitis. Identification of anti-NMDA receptor antibodies in serum and CSF confirmed the diagnosis.

In recent years, autoimmune etiologies of encephalitis are increasingly being recognized. Anti-NMDA receptor encephalitis has been regarded as the second most common immune-mediated cause, after acute disseminated encephalomyelitis, and is more prevalent than all antibody-associated encephalitis. This disease is a highly characteristic and recognizable neuropsychiatric syndrome, although atypical cases may exist [3–5]. It is most common in young women and children. Over half of the patients have an associated tumor, most commonly an ovarian teratoma [2].

Brain MRI in patients with anti-NMDA receptor encephalitis may be either normal or abnormal. The frequency of abnormal brain MRI is not as common as in infectious encephalitis such as herpes simplex encephalitis (HSE). It has previously been described that more than 90% of patients with HSE have MRI abnormalities, usually in the temporal lobe, frontobasal, and insular regions [6]. In a clinical-radiological comparison between herpetic encephalitis and limbic encephalitis of autoimmune etiologies (including anti-NMDA receptor encephalitis), MRI was abnormal in all cases of the former, but only in 60% of the latter [7].

In a cohort of 100 patients with anti-NMDA receptor encephalitis, brain MRI was unremarkable in 45 patients; in the other patients, T2 or FLAIR signal hyperintensity was seen in the hippocampi, cerebellar or cerebral cortex, frontobasal and insular regions, basal ganglia, and brainstem [8]. In another cohort of 44 patients, only 4 patients had abnormalities in the hippocampi, and 6 patients had abnormalities within white matter regions on T2/FLAIR [9].

The initial MRI of our case showed hyperintense lesions in bilateral subcortical white matter on FLAIR and DWI. FLAIR combined with DWI is more sensitive in detecting abnormalities than conventional MRI sequences. DWI has long been established as a routine imaging procedure in acute ischemic stroke. It is exquisitely sensitive in demonstrating even minute acute ischemic lesions [10]. It is now also used to diagnose other disorders including limbic system encephalitis. To our knowledge, multifocal subcortical white matter lesions on FLAIR and DWI have rarely been reported in previous literature, and serial MRI changes after plasma exchange have not been presented before. The clinical presentation of our patient, however, seemed unrelated to the lesions seen on FLAIR and DWI. Such abnormalities on MRI resemble that of demyelinating diseases such as acute disseminated encephalomyelitis, neuromyelitis optica, or multiple sclerosis. This may reflect autoimmune-mediated white matter disease. Literature suggests that anti-NMDA receptor encephalitis may have overlapping characteristics with other autoimmune diseases. For example, Zoccarato *et al.* described a case of anti-NMDA receptor encephalitis followed by neuromyelitis optica [11]. The coexistence of multiple autoimmune diseases is increasingly recognized among autoantibody-associated neurological syndromes [12].

The treatment of anti-NMDA receptor encephalitis includes immunotherapy and tumor removal, if applicable. As of yet, no randomized trials have been performed to evaluate the efficacy of immunotherapy. In a cohort of 577 patients (501 assessable), 472 underwent first-line immunotherapy (steroids, IVIg, or plasma exchange) or tumor removal, resulting in improvement within 4 weeks in 251 patients. Of the 221 patients who did not improve with first-line treatment, 125 received second-line immunotherapy (rituximab or cyclophosphamide), which resulted in a better outcome. Predictors of good outcome were early treatment and no admission to an intensive care unit [13]. For immunotherapy of anti-NMDA receptor encephalitis, Dalmau *et al.* prefer concurrent IVIg and methylprednisolone to plasma exchange because plasma exchange is more difficult to do in children, poorly cooperative patients, or patients with autonomic instability. If no response is seen after 10 days, second-line therapy should be initiated [14]. Other methods of immunotherapy have also been reported on rare occasions. Liba *et al.* described an 8-year old girl with anti-NMDA receptor encephalitis who had no response to sequential IVIg, plasma exchange and high-dose corticosteroids, rituximab, and cyclophosphamide treatment, but responded well to alemtuzumab, an anti-CD52 monoclonal antibody that affects memory B cells and T cells [15].

Our patient was initially treated with combined IVIg and methylprednisolone without demonstrable improvement. However, she responded well after plasma exchange was initiated. After several cycles of plasma exchange, she made a full recovery. Repeat detection of anti-NMDA receptor antibodies in serum and CSF were negative, and repeat MRI performed 1 month and 2 months after plasma exchange showed gradual resolution of the lesions.

## Conclusions

We describe a case of anti-NMDA receptor encephalitis concomitant with multifocal subcortical white matter lesions on FLAIR and DWI. This patient responded well to plasma exchange treatment, after which the lesions resolved.

## Consent

Written informed consent was obtained from the patient for publication of this case report and the accompanying images. A copy of the written consent is available for review by the editor of this journal.

## Abbreviations

Anti-NMDA receptor encephalitis: Anti-N-methyl-D-aspartate receptor encephalitis
CSF: Cerebrospinal fluid
MRI: Magnetic resonance imaging
FLAIR: Fluid-attenuated inversion recovery
DWI: Diffuse weighted images
anti-dsDNA: Anti-double-stranded DNA
ANCA: Antineutrophilcytoplasmic antibodies
anti-TG antibodies: Anti-thyroglobulin antibodies
anti-TPO antibodies: Anti-thyroid peroxidase antibodies
CT: Computed tomography
EEG: Electroencephalography
PCR: Polymerase chain reaction
IVIg: Intravenous immunoglobulin
HSE: Herpes simplex encephalitis
